# Ectothermic telomeres: it's time they came in from the cold

**DOI:** 10.1098/rstb.2016.0449

**Published:** 2018-01-15

**Authors:** Mats Olsson, Erik Wapstra, Christopher Friesen

**Affiliations:** 1 Department of Biological and Environmental Sciences, University of Gothenburg, Medicinaregatan 18, Box 463, 405 30 Gothenburg, Sweden; 2 School of Biological Sciences, The University of Wollongong, 2522 Wollongong, New South Wales, Australia; 3 School of Biological Sciences, University of Tasmania, Private Bag 55, Hobart 7001, Tasmania, Australia; 4 School of Life and Environmental Sciences, University of Sydney, Heydon-Laurence Bldg A08, Science Road, Sydney, NSW 2006, Australia

**Keywords:** telomeres, ageing, sexual selection, life history, reptiles

## Abstract

We review the evolutionary ecology and genetics of telomeres in taxa that cannot elevate their body temperature to a preferred level through metabolism but do so by basking or seeking out a warm environment. This group of organisms contains all living things on earth, apart from birds and mammals. One reason for our interest in this synthetic group is the argument that high, stable body temperature increases the risk of malignant tumours if long, telomerase-restored telomeres make cells ‘live forever’. If this holds true, ectotherms should have significantly lower cancer frequencies. We discuss to what degree there is support for this ‘anti-cancer’ hypothesis in the current literature. Importantly, we suggest that ectothermic taxa, with variation in somatic telomerase expression across tissue and taxa, may hold the key to understanding ongoing selection and evolution of telomerase dynamics in the wild. We further review endotherm-specific effects of growth on telomeres, effects of autotomy (‘tail dropping’) on telomere attrition, and costs of maintaining sexual displays measured in telomere attrition. Finally, we cover plant ectotherm telomeres and life histories in a separate ‘mini review’.

This article is part of the theme issue ‘Understanding diversity in telomere dynamics'.

## 1. Introduction

In a lecture on ‘Endothermy versus Ectothermy’ at University of Washington (2005), the distinguished physiologist and former ‘*Evolution*’ Editor Prof. Ray Huey divided the organismic world into two categories, birds and mammals (endotherms), and ‘the rest!’ (ectotherms). This paper is on telomeres and telomerase biology of ‘the rest’ [1,2]. This breadth should constrain how much could be said per taxon in a short review; however, there is a real dearth of research on the evolutionary ecology, genetics and physiology of ectotherm telomeres.

**Figure 1.**
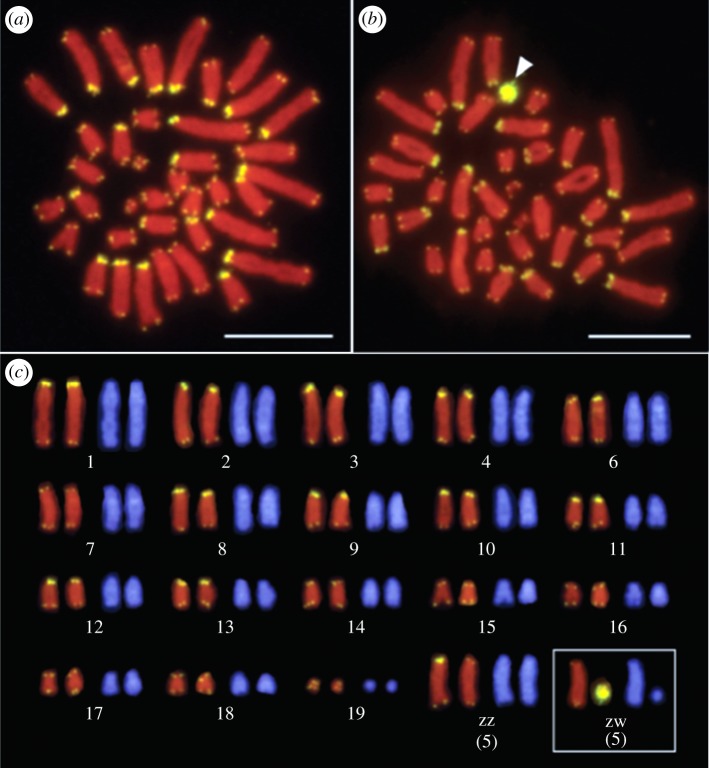
Chromosomal locations of the (TTAGGG)*n* repeated sequences in male (*a*) and female (*b*) *Lacerta agilis.* The arrowhead indicates the hybridization signals of the (TTAGGG)*n* sequence on the W chromosome. Scale bars represent 10 µm. (*c*) Full karyotype of *L. agilis* with telomeres as in (*a*) and (*b*) (Micrographs from Srikulnath *et al.* [5]). (Online version in colour.)

The non-coding DNA telomere sequences that are shielded by a protein complex are gradually lost with cellular age (and often chronological age) in many organisms [3] but are partly restored by telomerase [4], the reverse transcriptase coupled to an RNA template replacing the telomeric sequence (figure 1; TTAGGG/CCCTAA in all vertebrates [6]). Rarely, homologous recombination and copy switching also has a telomere lengthening effect (alternative lengthening of telomeres, ALT; [7]) but ultimately all loss of the telomere sequence at replication cannot be compensated for. At a critical stage of telomere shortening, the cell enters replicative senescence [6].

Most research on telomere length, its regulation by telomerase and telomerase somatic expression (and repression) has been performed on endotherms (mostly humans, e.g. reviews in [8–10] and references therein). In endotherms, as opposed to ectotherms, telomerase repression in somatic tissue, and telomere length distributions, have been suggested to be an evolutionary response to the risk of tumour development because of endotherms' higher metabolic rate and cellular replication. However, incidence of cancer (and its detection frequency) in nature remains poorly understood across organisms [11], including endotherms potentially with the exception of humans. It is clear, however, that cancer occurs throughout vertebrate and invertebrate taxa, both in the wild and in captivity ([12–16] but see [17] for potential exception in long-lived decapods). Specifically, there are considerable data from captive populations showing neoplasia in amphibians [18] and reptiles [19,20], and captive reptiles have been argued to have an incidence of neoplasia comparable with that of mammals and birds [16,21]. In fact, a study by Madsen *et al.* [20] demonstrates that reptiles in French zoo parks even have significantly *higher* cancer frequency than mammals. Similarly, cancer is widespread in fish, although malignant neoplasms with or without metastasis are reported ‘less commonly than in mammals’ [15]. Indeed, some amphibians (e.g. *Xenopus*) and fish (e.g. *Danio/Bracydanio rerio*, zebrafish) are used as models in cancer research specifically because they show high cancer frequency, regeneration, effects of regenerative tissue on cancer growth (negative), and because their tumours resemble human tumours both histologically and at a genetic level of expression [15]. While plants have orthologous tumour suppressors and oncogenes, mutations in these genes usually do not become cancerous and cell walls prevent metastasis [22]. The subject of cancer in ectotherms is clearly too large to cover in this review, and the data are still relatively meagre. However, we note that the risk of cancer has been claimed to be as high for ectotherms as for endotherms, suggesting that the postulated links between cancer, telomerase repression and endothermy/ectothermy are less straightforward than perhaps previously thought. In summary, ectotherms may come to play a key role in explaining ongoing evolution of telomerase repression in somatic tissue because—unlike most endotherms—they are likely to have variation in somatic telomerase expression, and associated telomere dynamics, that can be linked to corresponding variation in viability and fitness and be measured in real life.

How do we best understand the evolutionary ecology of telomere dynamics and telomerase suppression in ectotherms? Across Metazoa, there is wide variation in telomere dynamics with links to, for example, endothermy, ectothermy, regenerative ability of tissue and variation in telomerase production across tissue and taxa [9]. Thus, there appears to be an increasing acceptance and awareness that there is no single, universal pattern of telomere erosion and that our understanding has been restricted by studying primarily laboratory mammal models and humans with little or no telomerase production in somatic tissue. Even within taxonomic groups, such as ‘Reptiles’ (which admittedly is a synthetic, polyphyletic group that lumps taxa of highly diverse origin), telomere attrition patterns appear to be diverse. Alligators (Crocodilia), for example, show a decline in telomere length with age (closely related to birds; [23]), whereas much more complex patterns seem to be found in snakes and lizards [24,25], with increases and decreases of telomere length in relation to different telomerase production through life. Thus, in order to better understand ongoing selection and evolution of telomeres and telomere-regulating processes we need to incorporate work in the wild on non-model organisms that lend themselves better to research direct links between telomere traits and components of viability and fitness (table 1; box 1).

**Table 1.** Cited literature and taxa. GH, growth hormone; TL, telomere length.

reference	species	tissue(s)	method (telomere; telomerase if applicable)	telomere response and effect	telomerase?
Adriaenssens *et al.* [26]	wild juvenile brown trout (*Salmo trutta*)	fin and muscle	TRF	individuals with shorter fin telomeres to behave consistently more boldly and aggressively under controlled conditions in the laboratory. No such relationship was found with muscle telomere length 3–4 months after the behavioural assays	n.a.
Alibardi [27]	green anole lizard (*Anolis carolinensis*)	regenerating tail, testis, intestine	immunofluorescence and ultrastructural immunolocalization	n.a.	detected telomerase activity in regenerating tail tissues, developing spermatozoa
Anchelin *et al.* [28]	zebrafish (*Danio rerio*)	larvae; muscle and testis in adults	telomerase-deficient fish versus wild-type	TL shorter and quicker attrition in telomerase-deficient zebrafish	p53 was induced by telomere attrition, leading to growth arrest and apoptosis. Importantly, genetic inhibition of p53 rescued the adverse effects of telomere loss, indicating that the molecular mechanisms induced by telomere shortening are conserved from fish to mammals
Ballen *et al.* [29]	painted dragon lizard (*Ctenophorus pictus*)	blood	telomere PNA Kit/FITC for flow cytometry	maternal telomere length predicted offspring telomere length. Female reproductive investment was positively associated with offspring telomere length but offspring telomere length was negatively related to mitochondrial superoxide levels	n.a.
Carneiro *et al.* [30]	zebrafish (*Danio rerio*)	gut; testes; muscle;	TRF	decline in telomere length with age much stronger in tert- (telomerase-deficient fish); gut and muscle both exhibited decline, testes less so; DNA damage markers also correspond to telomere loss across tissues	telomerase-deficient fish show greater declines in telomere length with age across tissues
Gao & Munch [31]	Atlantic silverside (*Menidia menidia*)	pooled larval samples; muscle and brain tissue from adults	qPCR (melanocortin type 1 receptor (Mc1r) control gene)	no telomere decline with age; female fecundity was negatively correlated with telomere length and lifespan	n.a.
Giraudeau *et al.* [32]	painted dragon lizard (*Ctenophorus pictus*)	blood	qPCR (18s)	males that maintained colour suffered more telomere attrition	n.a.
Henriques *et al.* [33]	telomerase-deficient zebrafish (*Danio rerio*)	skin and fin	telomere repeat amplification (TRAP) assay	n.a.	yes using mutant lines
Joeng *et al.* [34]	nematode (*Caenorhabditis elegans*)	whole animals	TRF on transgenic lines that overexpress telomere binding protein (HRP-1)	worms with longer telomeres lived longer and were more resistant to heat stress	n.a.
Lund *et al.* [35]	zebrafish (*Danio rerio*)	heart, gills, kidney, spleen, liver, and intestine were evaluated at 3 months, 6 months, 9 months, and 2 years of age	TRF; TRAP	telomeres did not shorten with age in any tissue	all tissues and ages expressed telomerase
McLennan *et al.* [36]	Atlantic salmon (*Salmo salar*)	fin	qPCR (GAPDH control gene)	faster growth associated faster telomere attrition if they were exposed to harsher environment (predator density in stream)	n.a.
Näslund *et al.* [37]	brown trout (*Salmo trutta*)	fin	qPCR (GAPDH)	no effect of compensatory growth on telomere length; body size early in life was negatively related to telomere length later in life	n.a.
Olsson *et al.* [38]	sand lizard (*Lacerta agilis)*	blood	TRF	positive relationship between telomere length and age in females; negative but not significant in males. Tail loss had a stronger negative effect on telomere length in males than females	n.a.
Olsson *et al*. [39]	sand lizard (*L. agilis)*	blood	TRF	paternal age at conception predicts telomere length in sons; sire–son TL heritability is higher than mother–daughter; longer telomeres enhance offspring survival	n.a.
Olsson *et al.* [40]	sand lizard (*L. agilis)*	blood	TRF	females have longer telomeres than males; females suffer lower rates of attrition than males; telomere length had a positive effect on offspring recruitment in females but not in males	n.a.
Pauliny *et al.* [41]	coho salmon (*Oncorhynchus kisutch*)	fin	qPCR (beta-actin)	WT had shorter telomeres on both sampling occasions; but GH-fish had greater attrition; regeneration increased TL in GH-fish but not in WT	n.a.
Plot *et al.* [42]	leatherback turtle (*Dermochelys coriacea*)	blood	qPCR (18s)	no difference in TL between hatchlings and adults; breeding frequency of females was associated with shorter TL	n.a.
Rollings *et al.* [43]	mosquitofish (*Gambusia holbrooki*)	tail muscle	qPCR (GAPDH)	residual telomere length (TL | age in days) lowest in 20 < 30 < 20–30 = 30–20	n.a.
Rollings *et al.* [44]	garter snake (*Thamnophis sirtalis*)	blood	pPCR (18s)	TL was unchanged with age in females; TL decreased with age in males; TL was positively correlated with body condition in both sexes but body condition decreased with male age but increased with female age	n.a.
Tan *et al.* [45]	planarian flatworm (*Schmidtea mediterranea*)	whole animal (cultured)	TRF	telomere length in sexual animals decreases with age; telomere length in asexual animals increases after both fission and regeneration induced by amputation	The difference between sexual and asexual worms in telomere maintenance in due to the expression and alternate splicing of the protein subunit of the telomerase enzyme
Ujvari *et al.* [24]	frill-neck lizard (*Chlamydosaurus kingii*)	blood	qPCR (GAPDH); qPCR	TL increases with age until 4 years of age and then declines	positive relationship between TL and telomerase expression
Ujvari & Madsen [25]	Water python (*Liasis fuscus*)	blood	TRF	TL increased from hatching to 1 year of age and remained stable throughout life in males and females	n.a.
Walter *et al.* [46]	fruit fly (*Drosophila melanogaster*)	whole animal	strains with different telomere lengths	long telomeres associated with reduced fertility and fecundity	n.a.
Scott *et al.* [23]	alligator (*Alligator mississippiensis*)	blood	TRF	TL shorter in longer (and presumably older) animals	n.a.
Klapper *et al.* [47]	lobster (*Homarus americanus*)	hepatopancreas, heart, skin and muscle	TRAP	n.a.	telomerase expression in all tissues tested
Simide *et al.* [48]	Siberian sturgeon (*Acipenser baerii*)	fin and blood	qPCR (beta-actin)	decrease in TL with age and greater telomere attrition with heat stress	n.a.
Bronikowski [49]	garter snakes (*Thamnophis elegans*)	blood	TRF	decline in TL with age in males; females not studied nor were difference eco-morphs with different ageing trajectories	n.a.
Hatakeyama *et al.* [50]	medaka aka Japanese rice fish (*Oryzias latipes*)	embryo, whole body (1 day, 2, 3 6 months) liver, kidney, intestine, muscle, gonad, heart, brain, spleen, gill	TRF; TRAP	TL declines with age similarly among all tissues (except brain tissue) and is highly correlated between tissues. Telomere attrition was highest in developing stages	ubiquitous expression of telomerase across tissues
Gruber *et al.* [51]	marine clam (*Arctica islandica*)	gill, mantle, adductor muscle for all populations and foot, the heart, digestive gland in two populations	TRF; TRAP	although TL was variable it was not correlated with age or tissue type	consistently high telomerase activity that was not correlated with age
Debes *et al.* [52]	brown trout (*Salmo trutta*)	blood	qPCR(18s)	TL declines with increasing average summer temperature of the natal stream and with tail fork length (a proxy for body size)	n.a.
Nilsson *et al.* [53]	ascidian (*Diplosoma listerianum*)	zooids	telomere FISH; TRAP	telomeres were shorter in parents than sexually produced zooids	telomerase activity was lower in parents than sexually produced zooids
Schumpert *et al.* [54]	*Daphnia pulex* and *D. pulicaria*	whole animals	TRF and TRAP	TL is maintained throughout life in *D. pulex* (1–3 weeks) but declines in *D. pulicaria* (1–8 weeks)	telomerase activity is maintained in *D. pulex* but declines in *D. pulicaria* throughout life
Garcia-Cisneros *et al.* [55]	seastar (*Coscinasterias tenuispina*)	tube-foot	qPCR (no SC control gene ‘telomeric DNA measurements in the present study were performed relative to the total quantity of DNA in the samples’)	telomere length was longer in individuals from clonal populations and longer in regenerating arms than non-regenerating arms	n.a.
Korandova & Frydrychova [56]	honey bee (*Apis mellifera*)	embryos, brain, testes	TRF; TRAP	no difference in telomere lengths in any comparisons (tissue; hive; castes)	telomerase levels high in workers and drones at embryo stage; high in drone testes; high in brain and ovaries of queens
Bousman *et al.* [57]	African clawed frog (*Xenopus laevis*)	skeletal muscle, heart brain, liver, lung, spleen, testis, embryo	TRAP	n.a.	telomerase was most highly expressed in testis, spleen liver and embryos; detectable in muscle and brain

Box 1. **Telomere biology in plant ectotherms.**

‘I shall publish in a month or two a book on the ‘Movement of Plants’. I will send you a copy, but I fear it is much too special for anyone but a physiological botanist to care about. I have long thought that old men, like myself, ought to write only on confirmed & easy subjects.’

Charles Darwin, Oct. 7, 1888

(unpublished letter to Ernst Krause)

This father and son volume by Charles and Francis Darwin [58] captures with characteristic and remarkable insight many of the traits in plants we often see as uniquely ‘zoological’, such as ‘sleeping’, when plants get a thermoregulatory advantage from drooping their leaves as opposed to keeping them horizontal at night. Not ‘sleeping’ would expose them to the clear night sky, Darwin speculated, and experimentally verified that ‘sleeping’ resulted in a significant reduction in frost damage (92% of sleepers survived, whereas only 37% of controls did; statistically verified difference by Huey *et al.* [59]).

**Figure 2.**
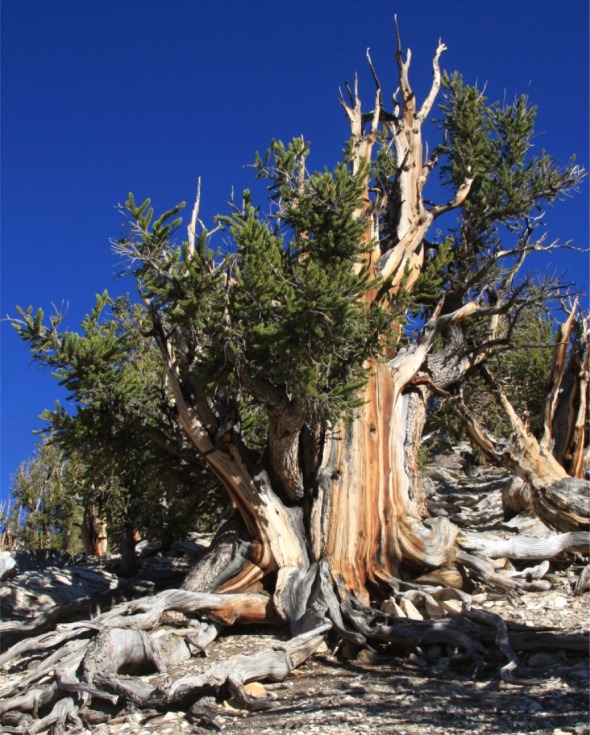
*Pinus longaeva*, Bristlecone pine, is one of the oldest pine trees, with specimens recorded as over 5000 years of age. (Online version in colour.)

‘Ectothermic organisms’ also include plants, and it seems appropriate to first introduce them here in a separate section, since this issue is primarily zoological. Plants have the widest range of lifespan of all eukaryotes (1 year in annuals to more than 4000 years in single species, to more than 10 000 years in some clonal species [60,61]). However, there is a dearth of studies of the evolutionary ecology of plant telomeres. Plant longevity is principally determined by the indeterminate growth of vegetative meristems [62]. The totipotency of meristems through the organisms' life can, in some cases, ameliorate the telomere shortening that is typical of cells that do not express telomerase [63]. The oldest known non-clonal organism is a bristle cone pine (*Pinus longaeva*) at nearly 5000 years old (figure 2). Indeed, bristle cone pines do not exhibit declines in function that characterize analogous senescence in animals (e.g. water/nutrient transport, pollen viability, or seed germination [64]). Bristle cone pines also do not exhibit age-related decline in telomere length in needles, and telomeres may even increase slightly in root cells [65]. In another long-lived non-clonal tree, *Gingko biloba*, telomeres seem to increase early in life (10, 20, 70 through 100 years of age) and then remain stable through to 700 years of age (oldest trees in the study), maintaining some telomerase expression over this same age range [66]. Interestingly, telomerase activity in Gingko is highest in tissue undergoing repair/regeneration (i.e. embryonal callus) and during flowering in microspores (sex cells) compared with leaf tissue. Indeed leaf tissue showed a seasonal-expression pattern with the highest activity during leaf formation and growth and a decline to eventual leaf-drop in the autumn [66]. Given the age ranges included in these studies, these are necessarily cross-sectional data, which require circumspect interpretation as it is possible that selection leaves only the trees with these properties standing. Furthermore, studies in other land plants indicate no or little telomerase activity in vegetative tissue similar to humans but do exhibit telomerase expression during flowering, early seasonal growth and germination [67,68].

Telomeric sequences of plants are more diverse than in metazoans. Although TTTAGGG is the most widespread telomeric sequence in the land plants and the green algae, there are at least four different telomere motifs in the land plants and at least another two unique sequences in green algae [69], including the familiar TTAGGG found in vertebrates. The mechanistic biology governing telomere dynamics (i.e. attrition and elongation) in plants and animals is very similar, although there is divergence in protein complexes associated with telomeric sequences serving analogous functions [62,69]. Telomere length is maintained at an ‘optimal’ length by the action of telomerase and alternative lengthening and by dissociation of telomere binding proteins, which then exposes them to endogenous nucleases that shorten them [69].

The most salient and critical function of plant telomeres and their associated proteins is to maintain genomic stability, prevent endogenous DNA erosion at chromosome termini and aberrant chromosomal rearrangements [70,71]. Nevertheless, plants have an amazing capacity to withstand genomic instability generated by severely shortened telomeres [68,72] evading telomeric fusions even when telomeres shorten to less than 1% of their normal length [73]. TERT-mutant lines of *Arabidopsis* that are unable to maintain telomere length via telomerase activity survive and successfully reproduce for 10 generations, although with increasing cytogenetic impairment and eventual vegetative arrest and reproductive senescence [74]. The role of telomeres in whole plant ageing is more controversial (see [60], and [62] for contrasting views on the subject). However, telomere and other DNA damage during seed ageing are main determinants of germination success [75]. During imbibition, there is significant upregulation of telomerase and DNA repair genes [76].

Although, as Darwin indicated, plants are not behaviourally inert, plants are sessile and thus have fewer options to escape the vagaries of daily and seasonal environmental conditions. Most structures and organs in plants are formed by plastic proliferation of meristematic cells that continue throughout adult life, which results in indeterminate growth. This is very different from animals, such as fish, snakes and lizards, that also exhibit indeterminate growth. The modular (i.e. root, stems and leaves) phenotypic plasticity of plants, saddled with an evolutionary history of ‘toughing it out’ exposed to the elements, may explain the huge difference in plant age and telomere dynamics compared with animals. Plant structures are dispensable. For example, leaves are shed and in woody plants and trees the core is made up of mostly dead cells. Perhaps this is, in addition to having cell walls, why plants are not known to exhibit metastasized neoplasia [22]. These transitions from live to dead cells are programmed such that resources are recycled and stored for next season's growth (in perennials) or packaged in resilient seeds that sprout the next generation in annuals. Plants, unlike animals, do not have a defined germline, gametogenesis occurs late in tissue development. In theory, somatic mutations can be transmitted to the next generation as germlines. However, in *Arabidopsis*, germline cell divisions are independent of both plant age and vegetative age [63], meaning older plants do not pass along longer or shorter telomeres. Tracing telomeres through successive generations, [63] found that the shortest telomeres were typically elongated in the subsequent generation, while the longest telomeres were usually shortened.

An important aspect of many research projects in evolutionary biology is the estimation of coefficients of selection acting on traits, which is the covariation between relative fitness (most often measured as lifetime reproductive success, LRS) and individual trait measures [77]. Selection coefficients multiplied by the narrow-sense heritability formally depicts the evolutionary response. In telomere biology, the effect of reproduction as a component of selection has rarely been quantified (most often focusing on survival but ignoring reproductive output as part of the selection pressure). Thus, research on telomere dynamics should involve selection estimates taking reproduction into account, that is acting before selection ‘goes blind’. Thus, we have little fundamental understanding of ongoing evolutionary processes that dictate telomere evolution in the wild. Insight into how variation in telomere length and telomerase production in ectotherms covary with significant drivers of life-history evolution and lifespan would thus be of considerable interest. For example, in many ectotherms there is no, or very little, reproductive senescence in healthy animals and fecundity increases with body size and age (e.g. [47,78]). However, when disease occurs early in life, prior to or during reproduction, it affects lifetime fitness and is a component of natural selection [79]. Thus, in some ectotherms at least, selection on disease operates over a greater proportion of an individual's life than occurs in many mammals and especially in humans. Links between early- and late-life telomere length, their association with growth and cancer risk, thus seem particularly important in many ectotherms. Gerontological and epidemiological arguments in the perspective of evolution of lifespan merge together [80], since disease incidence increases with age for many, but not all diseases.

One of the challenges in understanding telomere dynamics remains assigning cause and effect, especially in systems where an experimental approach is not possible. In non-experimental research on telomeres, and their covariation with other genotypic and phenotypic traits, trait categories can be assigned response or predictor variable depending on researcher preference (or bias). For instance, relatively longer telomeres (on the *X* axis) may be predicted to causally result in relatively higher reproductive output (e.g. clutch size). On the other hand, the argument can be turned on its head and large clutch size (on the *X* axis) can be argued to result in telomere attrition as a cost of reproduction. What is true is up to the interpreter and other supporting data. Furthermore, researchers have had very few means to manipulate telomeres *per se*. If telomeres were eroded using reactive oxygen species (ROS; [81]), or rescued from those using antioxidants [82], effects on other life-history parameters, or cell–cell signalling, are likely to compromise the experimental outcomes and interpretations. The use of telomerase knock-down mutants provides a potentially powerful way to understand the role of telomerase in telomere dynamics, ageing and disease processes in both ectotherms and endotherms.

Here, we provide an integrative synthesis of telomere dynamics in ectotherms. We focus on (a) life history and telomeric covariation, (b) telomere links to personality, predation and proliferation, and (c) telomere length: selection and heritability.

## 2. Life histories and telomeric covariation

Many ectotherms have served as excellent models of telomere research, some concerning classic life-history questions, such as ageing and the reproduction–somatic maintenance trade-off. As individuals age and accumulate costs of living and reproduction, telomeres are predicted to shorten concomitantly. Growth in ectotherms is more plastic in response to environmental drivers (especially temperature) than in endotherms, providing the opportunity to tease out the costs of growth and age effects on telomere attrition. Below we detail the links between age, growth, reproduction and telomere dynamics.

### (a) Links to age and growth

**Figure 3.**
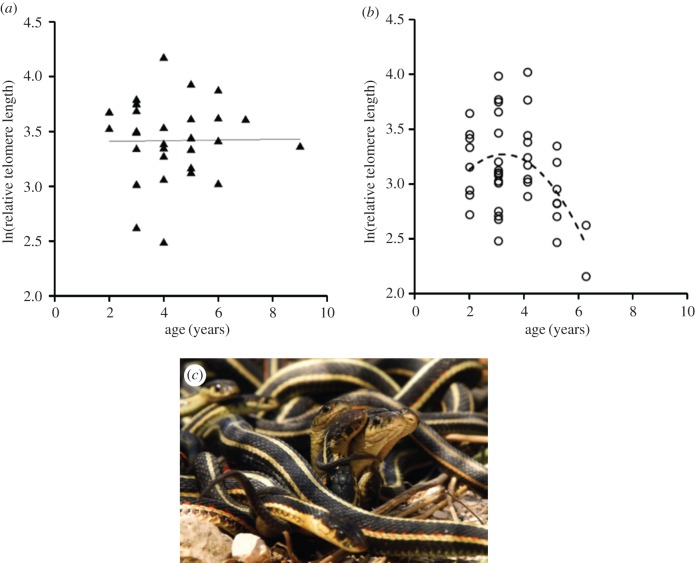
Sex differences in age-dependent telomere length with no relation in (*a*) females, but a curvilinear decline of telomere length with age in (*b*) males in the red-sided garter snake (*Thamnophis sirtalis parietalis*). Females show a much less active reproductive strategy. In picture (*c*), a passive female (the large, central head) is surrounded by smaller heads of energetically courting males. (© CRF, From Rollings *et al*. [84]). (Online version in colour.)

Growth in ectotherms is the outcome of synergy between a complex set of drivers, such as temperature and innate capacity for growth set by acclimation processes, and the accumulated damage from ageing ([83]; for instance, growth may change differently with temperature due to past selection in different thermal environments). For example, a recent study by Simide *et al.* [48] showed telomere shortening with age in the Siberian sturgeon (*Acispenser baerii*) using both longitudinal and cross-sectional sampling. A study on garter snakes (*Thamnophis elegans*) verified similar cross-sectional relationships between age and telomere length ([49]; figure 3). Other work has, however, shown no, or far less, such age effects on telomere length ([41] for Atlantic salmon, *Salmo salar*, [40] for sand lizards, *Lacerta agilis*). Work on another reptile, the leatherback turtle (*Dermochelys coriacea*), demonstrates that there is no difference in telomere length between hatchling and old turtles, suggesting a high telomerase activity early in life [42]. Hatakeyama *et al.* [50] showed age-specific telomere shortening in the freshwater teleost (*Oryzias laticeps*) despite both ubiquitous and lifelong telomerase activity. In a later study on the same species, however, the same group showed that telomerase activity varied through life (lower activity early and late in life), with corresponding shifts in telomere length [85].

Ectotherms also provide some of the most extreme examples of lifespan and thus relationships with telomere length, potentially becoming an interesting life-history component. For example, in the longest-living non-colonial animal in the world, the bivalve, *Arctica islandica*, there is no relationship between telomere length and age (or telomerase activity) despite ages in excess of 200 years [51]! In bristle cone pines (*Pinus longaeva*), the longest-lived non-clonal plants, telomere length is stable throughout its approximately 5000 year life ([65]; figure 2).

Ectotherms potentially provide a powerful way forward to understand the dynamics between age and size, and telomere length regulation, with experimental manipulation of growth and a concomitant examination of changes in telomere length or telomerase activity. In most ectotherms, growth is indeterminate (although it declines with age and size [78]) and should, ideally, be manipulated while leaving other systemic parameters, such as size and body temperature, unaffected. Very few systems allow for such experimentation but a clever way around these problems was an approach by Jörgen Johnsson's group, who manipulated growth hormone (GH) levels in salmonids by comparing transgenic fish (with doubled-up GH genes). Fish with extreme growth rates were compared to wild-type fish in a split brood design, which revealed that GH-manipulated fish suffered much higher telomere attrition [41]. In plants, vegetative growth, size, reproduction and lifespan can be manipulated via modulating day length. This technique was used to test and confirm that the number of mutations and telomere changes that were transferred to the next generation was constant, regardless of parental age and length [63].

Ectothermic growth is strongly dependent on the thermal environment and this can be manipulated to alter growth rate, and potentially telomeric length trajectories. For example, not only do telomeres shorten with age in the Siberian sturgeon (*Acispenser baerii*), but Simide *et al.* [48] also induced a 15% telomere loss over a single month using heat treatment (30°C). How this comes about is not straightforward. We found no published data on erythrocyte lifespan in sturgeons but, assuming it is similar to that in carp (*Carassius auratus langsdorfi*)—51 days on average [86]—telomere loss should come about through double- or single strand breaks in erythrocytes rather than proliferation of haematopoietic stem cells in the bone marrow (and strand break ill-effects would be hard to differentiate from other more genome-wide effects [81]). An alternative explanation to changes in telomere length distribution would be biased mortality of cells with longer telomeres (although this runs counter to the fact that shorter, not longer, telomeres induce apoptosis). Similar effects could explain telomere shortening at relatively higher temperature in wild brown trout (*Salmo trutta*; [52]). In contrast, colder (harsher) environments could cause corresponding telomere shortening, as shown in other brown trout populations; thus, telomere length is assumed to be optimized by stabilizing selection [36,87]. Interestingly, and contrary to expectations, part of this stabilizing selection scenario seems to be increased survival at sea by successfully migrating and returning salmon with shorter telomeres [88]. Temperature affects growth rate, and hence cell division and proliferation, so a straightforward prediction is that telomere attrition should be higher in warmer water, and in particular in water warmer than where selection took place. Rollings *et al.* [43] explored catch-up growth effects in mosquito fish (*Gambusia holbrookii*) but only found a weak difference among treatment groups, with fish in constant 20°C having shorter telomeres than in treatments with fish experiencing a gradual change from 30°C to 20°C. Similarly, Näslund *et al*. [37] assessed compensatory growth in brown trout and found no increase in telomere erosion at elevated growth rate. These divergent results are presumably explainable by differential gene expression of telomerase production depending on temperature. In human cell culture, thermal treatment (37, 39, 42°C) causes a shift in telomere length distribution towards mid-length telomeres, but with no temperature effect on telomerase production [89]. Thus, telomere attrition (caused by ROS production) and restoration (by telomerase upregulation) may not be linearly dictated by temperature changes or even work in the same direction with the same change in temperature. Thus, temperature-dependent (nonlinear) up- and down-regulation of telomerase expression needs to be examined, independent of telomere elongation and attrition, for a more complete understanding of telomere dynamics.

Promising research in which metabolic processes are experimentally altered in ectotherms shows ‘dynamic dynamics’ of telomere regulation and potentially environmental dependence. This agrees with recent work in endotherms that are acting like ectotherms, that is, work exploring variation in telomere length with torpor and hibernation in small rodents. Turbill *et al.* [90] showed that in the edible dormouse (*Glis glis*) telomere attrition was arrested during hibernation, supporting the idea that torpor slows ageing and might be responsible for relatively longer life in this and other species that hibernate. Subsequent work on the same species by Hoelzl *et al.* [91], however, showed that hibernation is not without cost; arousal from hibernation (especially repeated arousal) was associated with telomere shortening, potentially through oxidative stress. Turbill *et al.* [92], this time working on Djungarian hamsters (*Phodopus sungorus*), tested the hypothesis that torpor slows ageing in this highly seasonal rodent that uses daily torpor and found that relative telomere lengths increased in individuals that undertook frequent bouts of daily arousal. An interesting comparison would be similar work on truly ectothermic animals, such as reptiles, that undergo similar periods of hibernation, aestivation and daily torpor.

Knock-down or knock-out telomerase mutants or genetic lines within species with fast versus slow growth potentially offer mechanisms to further understand the relationship between telomerase activity and telomere length in ectotherms. Anchelin *et al.* [28] showed the value of zebrafish telomerase knock-down mutants for studies of ageing, with an increase in short telomeres leading to growth arrest and apoptosis and other ageing symptoms such as spinal curvature, liver and retina deterioration and infertility [28]. This agrees with the work by Joeng *et al.* [34], who showed that lifespan in nematodes (*C. elegans*) is strongly prolonged (10–19.3%) by experimentally elongating telomere length, but that the effect depends on the *daf*-*16* gene. These effects were dismissed by Simons [93] as causally affecting lifespan, but here we acknowledge that such interaction effects are causally important even if indirect, since epistatic and regulatory effects are also evolutionarily relevant. Schumpert *et al.* [54] took advantage of two closely related ecotypes in *Daphnia* to explore the relationship between growth, telomerase and telomere lengths. In the short-lived ecotype (*D. pulex*) there was no age-dependent decline in telomere length or telomerase activity, while in contrast there was a significant age-dependent decline in telomere length and telomerase activity in the longer-lived *Daphnia pulicera*. How well these patterns hold up under increased scrutiny of more ectothermic species remains to be seen (see [94] for a cross-species comparison of telomere shortening in slow-ageing versus fast-ageing species).

### (b) Links to reproductive modes, sexual dimorphism and polymorphism

Telomere regulation is predicted to vary with reproductive mode (e.g. sexual versus asexual reproduction) and with reproductive effort. Where energetic commitment to growth versus reproduction differs between the sexes and this leads to sexual size dimorphism, sexual differences in telomere dynamics are predicted. Whereas sexual animals achieve telomere elongation through sexual reproduction, asexuals maintain telomere length by fission or when regeneration is induced by amputation. In organisms that propagate by agametic cloning, the parental body is the reproductive unit and fitness increases with clonal size. Therefore, clonal metazoans have been considered near ‘immortal’ [53]. Recent work on clonal ascidians shows that the passage between sexual generations provides total rejuvenation permitting indefinite propagation and growth and that parents have strikingly lower levels of telomerase compared to their offspring; thus, parents seem to ‘run out’ of telomerase as a result of reproduction compared to their offspring [53]. However, in other species (fissiparous starfish *Coscinasterias tenuispina*), clonality is associated with longer telomeres, potentially mediated by population-specific telomerase expression [55]. Similarly, in planarian flatworms (*Schmidtea mediterranea*), somatic telomere maintenance is different in asexual and sexual animals mediated by telomerase expression; asexual animals maintain telomere length somatically during reproduction by fission or when regeneration is induced by amputation, whereas sexual animals only achieve telomere elongation through sexual reproduction [45].

Ageing effects and costs of reproduction in more fecund individuals would be expected to covary inversely with lifespan and are also predicted to affect telomere dynamics. This has been shown in Atlantic silversides (*Menidia menidia*; [31]) where more fecund fish had shorter lifespans (as predicted by classical life-history theory) and also had shorter telomeres. When such trade-offs in investment patterns between growth, lifespan and reproduction differ between the sexes, the evolutionary outcome may be sexual dimorphism. As a corollary, the sexes may also differ in telomere traits; if telomeres shorten with cell division and one sex grows faster than the other, or differ in some other aspect of reproductive investments, sexual dimorphism in size may co-occur with that in telomere length ([95] and references therein). Ongoing differential selection on body size between the sexes, and corresponding selection in the wild on telomere length, have been demonstrated in a (correlative) quantitative genetics field study of sand lizards [40]. Such selection processes could lead to the evolution of the drastically different life-history strategies in the sexually size-dimorphic red-sided garter snake (*Thamnophis sirtalis parietalis*; [44]), where females grow much larger and live longer than males. Male and female red-sided garter snakes also differ in their corticosterone levels (higher in males) following hibernation, with males having costly mating behaviours while females have a much more passive role and reproduce biennially. Telomere erosion is predicted to be more pronounced in males than females, and this indeed proved to be the case; males had a (negative) quadratic telomere length decline with age, whereas females maintained their telomeres without noticeable attrition ([44]; figure 3).

**Figure 4.**
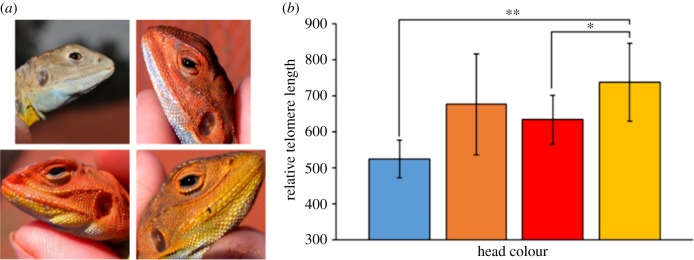
(*a*) The four male morphs of painted dragons (*Ctenophorus pictus*). (*b*) Mean (±s.e.) relative telomere lengths (RTL) of the four male morphs. RTL of yellow males was significantly higher than red and blue males (from Rollings *et al*. [44]; see text) (© CRF). (Online version in colour.)

Telomere length and attrition are believed to reflect aspects of the ageing process [96], which is linked to fitness. If so, then exterior, phenotypic traits may act like ‘health certificates’ to partners and rivals, covary with telomeres and be under sexual selection. Giraudeau *et al.* [32] investigated the relationships between colour fading during the mating season and telomere attrition in Australian painted dragon lizard (*Ctenophorus pictus*). They concluded that levels of ROS at the onset of the mating season were unrelated to initial telomere length, but those lizards that better maintained their coloration also lost more telomere bases [32]. Furthermore, work on the same species used its polymorphism with differences among morphs in head colour and associated reproductive behaviours, including level of aggression and investments into reproduction. In accordance with predictions, morphs with high investment strategies into reproduction also had shorter telomeres, which captures the relatively lower levels of somatic maintenance ([84]; figure 4). In an extreme example of intraspecific life-history polymorphism, the honeybee, *Apis mellifera*, Korandova & Frydrychova [56] reported that telomerase activity and telomere length were regulated in a development- and caste-specific manner, and highly variable among castes, with queens exhibiting the greatest telomere lengths and concomitant telomerase expression.

Thus, high-level ornamentation seems to be costly and condition-dependent. In the red-sided garter snake, telomere length was positively correlated with body condition in both sexes, but overall males had much lower body condition than females and it decreased with age in males, a pattern that was mirrored in telomere loss [44].

## 3. Telomere links to personality, predation and proliferation

Other drivers of telomere dynamics appear to be individual behaviour and ‘the pace of life’ [97–99]; this is likely to have significant effects in a range of physiological processes, including telomere attrition. A study of brown trout [26] showed that bolder, more aggressive and exploratory personalities had shorter telomeres. Ectothermic taxa thus provide an opportunity to explore the effect of tissue regeneration on telomere dynamics. For example, in many lizards tail autotomy is used to reduce predation but has energetic costs during tail regrowth [38]. During this period, a more cryptic lifestyle is adopted and bodily growth is stunted in favour of tail regrowth. In sand lizards, this comes at a cost in terms of telomere length but only in males (not in females that are camouflaged) and more so in the less visual, smaller, younger males [38], suggesting a further hidden context-dependent cost to autotomy and tissue regeneration. How are these rapidly replicating cells protected from the effects of telomere shortening, especially considering that tail loss can happen repeatedly during an individual's life? Immunolocalization techniques suggest that telomerase is upregulated in the cells of lizard tails after autotomy [100]. This mechanism may be widespread in ecothermic taxa; in a variety of plants, marine invertebrate and vertebrate ectotherms, telomerase activity is upregulated and telomere lengths maintained during regeneration, in some cases preferentially in the shortest telomeres being elongated [66,101,102].

In zebra fish, telomeres shorten to critical length only in specific tissues and independently of their proliferation rate [30]. Short telomeres accumulate at the same rate in the (highly proliferative) gut, and muscle (low proliferative), and indicate age-associated disease, including cancer, before these become phenotypically noticeable [30]. Telomerase production is lifelong in all zebra fish somatic tissue and telomere length is maintained through life in all tissue, too [35]. Amphibian telomeres seem to be the least explored of the vertebrate groups. However, in classic model systems, such as *Xenopus* (*X. laevis* and *X. tropicalis*), all tissues examined contain active telomerase, and most abundantly in the testis, spleen, liver and in the embryos [57]. Again, however, there are different patterns in different ectothermic taxa; in the cockroach (*Periplaneta americana*) telomerase is upregulated in young instars and gradually declines during development, has differential tissue activity and is most active in the testes and ovaries [103]. In lobsters (*Homarus americanus*), which have indeterminant growth, high telomerase activity was found in all major organs and was argued to be responsible for maintaining long-term cell proliferation and for preventing senescence [47].

## 4. Telomere length: selection and heritability

In analyses of evolutionary responses to selection, studies of heritabilities are combined with the estimation of selection coefficients [77]. The few studies of wild ectotherms, however, show considerable differences in the relationship between telomere traits and survivorship or lifespan. Ujvari & Madsen [25] refuted telomeric effects on fitness in tropical pythons (*Liasis fuscus*). In that study, telomere length increased early in life but then asymptotically levelled out in older age [25], and because of sex-specific growth trajectories, this resulted in longer telomeres in females than males. No relationship with survivorship was found [25]. A second study by Ujvari *et al.* [24] on frill-neck lizards (*Chlamydosaurus kingii*) in tropical Australia showed short telomeres in young lizards, then telomere growth in midlife, followed by attrition in older lizards.

How does telomere length at the cellular level correlate with cell viability and evolutionary fitness? These questions remain relatively poorly understood. An example of potential threshold effects of telomere length on cell viability are Ujvari *et al.*'s [24] studies on frill-neck lizards, in which they conclude: ‘telomere length dynamics reflect an adaptation to maintain telomere length above a critical minimum in order to maintain cellular homeostasis’. Telomeres signal at a critical minimum length and cell-suicidal apoptosis can be an outcome of this process. However, many studies in our review confirm covariation between telomere length and attrition, and covariation with components of fitness. This is not predicted by a critical, minimal telomere length effect. Thus, the idea of a ‘critical minimum’ telomere length fails to explain broad, quantitative patterns of covariation with components of fitness in evolutionary ecology and genetics. One such pattern between telomere attrition and costly maintenance of breeding colours seem to be the case of the painted dragon lizard (*Ctenophorus pictus*). In this species, maternal telomere length was a predictor of offspring telomere length and was significantly heritable despite statistical effects of oxidative stress on telomere length [29]. Heritability estimates of telomere length in wild sand lizards was more than one (1.23) for son–sire heritabilities, and 0.55 for daughter–dam estimates in the wild [39]; heritability of telomeres, like any statistically sampled trait, can show values larger than one [77]. Furthermore, it is important to note that for sand lizards, there was no environmental confound of the regression effects of mean offspring on parental telomere length because the offspring were released at random at the study site for over a decade [104]. These results seem to suggest a more complex inheritance pattern of telomeres than a simple Mendelian process [39]. For example, Olsson *et al.* [39] showed that paternal age is negatively correlated with offspring telomere length, suggesting an epigenetic, transgenerational effect through which telomere sperm shorten through life, resulting in a negative paternal age effect on son telomeres (unlike in some mammals [105]; see also Postma [106] for a detailed review on choice, suitability and differences in outcome of different methods for analysing heritability and components of additive components of variance).

An important, but often overlooked, relationship is whether selection and epigenetic effects of telomere length in adults can act via genetic interactions between life-history stages [107], so that hatchling telomere traits are affected by selection on adults via genetic correlations. Thus, selection on adults could in this way causally impact probability of survival and, ultimately, lifespan also at a juvenile life-history stage. The best evidence comes from experimental work on the offspring of transgenic nematodes (*C. elegans*), non-transgenic worms in the F1 generation retained lifespan-extending effects from elongated telomeres, but the effects were dependent on *daf-16* for lifespan extension in the F1 phenotype [34].

## 5. Conclusion and future prospects

With the exception of phylogenetic descriptions, and molecular mechanisms of telomere dynamics in models such as *C. elegans* (a nematode), *Saccaromyces cerevisiae* (Baker's yeast) and *Arabidopsis*, there is a real dearth of research on the evolutionary ecology, genetics and physiology of ectotherm telomeres. However, with the increasing number of studies that are becoming available on non-model organisms, it is also becoming increasingly clear that there is no single, universal pattern of telomere dynamics. Telomerase, the main driver of telomere elongation, occurs throughout the body in, for example, many fish, and associated with this is an approximate equal telomere length across tissue. Future work in evolutionary ecology on telomeres needs to embrace work on non-model taxa in natural populations, incorporating the effects of telomere traits on reproductive parameters. In particular, species with telomerase production in somatic tissue may be suitable models for understanding selection pressures from disease susceptibility (e.g. cancer) associated with variation in telomerase production and associated variation in cancer risk. Thus, ectotherms are likely to be a treasure chest for studies of ongoing evolution of telomeres and telomerase dynamics in the wild and may hold the key to understanding ongoing selection on variability in somatic expression and evolution of somatic telomerase repression.

## Data accessibility

This article has no additional data.
